# Activation of GPR35 protects against cerebral ischemia by recruiting monocyte-derived macrophages

**DOI:** 10.1038/s41598-020-66417-8

**Published:** 2020-06-10

**Authors:** Ozayra Sharmin, Ariful Haque Abir, Abdullah Potol, Mahabub Alam, Jewel Banik, A.F.M. Towheedur Rahman, Nuzhat Tarannum, Rasiqh Wadud, Zaki Farhad Habib, Mahbubur Rahman

**Affiliations:** 1 Laboratory of Pharmacology, Department of Pharmaceutical Sciences, School of Health & Life Sciences, North South University, Bashundhra R/A, Dhaka, 1229 Bangladesh; 2 Present Address: Faculty of Medicine, Friedrich Schiller University Jena, 07743 Jena, Germany; 3 Present Address: Deptartment of Neurobiology & Developmental Sciences, College of Medicine, UAMS, 4301W. Markham St., Little Rock, AR 72205 USA; 4 Present Address: Milwaukee Institute of Drug Discovery, Department of chemistry and Biochemistry, University of Wisconsin-Milwaukee, Milwaukee, WI 53211 USA; 5 Present Address: Department of Veterinary Medicine, University of Cambridge, Madingley Road, Cambridge, CB3 0ES UK; 6 Present Address: Department of Physiology, Development and Neuroscience, University of Cambridge, Downing Street, Cambridge, UK

**Keywords:** Diseases of the nervous system, Neurodegenerative diseases, Stroke, Neuroscience

## Abstract

Pamoic acid is a potent ligand for G protein Coupled Receptor 35 (GPR35) and exhibits antinociceptive property. GPR35 activation leads to increased energy utilization and the expression of anti-inflammatory genes. However, its role in brain disorders, especially in stroke, remains unexplored. Here we show in a mouse model of stroke that GPR35 activation by pamoic acid is neuroprotective. Pharmacological inhibition of GPR35 reveals that pamoic acid reduces infarcts size in a GPR35 dependent manner. The flowcytometric analysis shows the expression of GPR35 on the infiltrating monocytes/macrophages and neutrophils in the ischemic brain. Pamoic acid treatment results in a preferential increment of noninflammatory Ly-6C^Lo^ monocytes/macrophages in the ischemic brain along with the reduced neutrophil counts. The neuroprotective effect of GPR35 activation depends on protein kinase B (Akt) and p38 MAPK. Together we conclude that GPR35 activation by pamoic acid reprograms Ly-6C^Lo^ monocytes/macrophages to relay a neuroprotective signal into the ischemic brain.

## Introduction

G protein Coupled Receptors (GPCRs) are the largest family of cell surface receptors that play a critical role in regulating many important physiological functions. Stimulants such as hormones, light, lipids, odorants, and neurotransmitters activate these receptors^1^. Upon activation, GPCRs modulate diverse intracellular signaling pathways. Therefore, GPCRs are successfully targeted for many therapeutic approaches, including neurodegenerative disorders and stroke^2,3^. While conducting a human genomic DNA screen, G protein Coupled Receptor 35 (GPR35) was discovered^4^. It is a rhodopsin-like, Class A GPCR that is expressed in the CNS and peripheral nervous system in a region-specific manner. For instance, mouse medulla oblongata, hippocampus, spinal cord, and dorsal root ganglia (DRG) express GPR35^5^. Human caudate nucleus and DRG express GPR35. The spinal cord, hippocampus, and cerebrum of rats have been reported to express GPR35^1,6^. Immune cells including monocytes, dendritic cells, peripheral blood lymphocytes, neutrophils^7^, and natural killer cells, highly express GPR35^8^. These indicate the potential involvement of GPR35 in the immune modulation of the nervous system^9^.

GPR35 signals via Gα_i/o_ pathways^10^. It also mediates its function through Gα_13_ and β-arrestin-2^11^. Gα_i/o_ signaling has been reported to interact with extracellular signal-regulated kinase 1/2 (ERK1/2), protein kinase B (AKT), and p38. These signaling molecules are critical in determining stroke outcome^12,13^. GPR35 shares the closest homology with the purinergic receptor LPA4 (32%), and the hydroxycarboxylic acid receptor HCA2 (30%)^9^. Interestingly, HCA2 is also a GPCR that is expressed on immune cells including neutrophils, monocytes, and macrophages and has been reported to be neuroprotective in stroke and multiple sclerosis^2,3^.

Pamoic acid (PA) is a potent GPR35 agonist that exhibits an antinociceptive property mediated through GPR35^14^. In contrast, pamoic acid is considered to be inert^15^ and currently in use to improve the dissolution of pharmaceutical formulations^16^. GPR35 activation by pamoic acid may increase the phosphorylation of ERK1/2, which in turn initiates an anti-inflammatory signal by suppressing NF-κB-dependent inflammatory genes^17^. Activation of AKT signaling by pamoic acid through GPR35 may critically involve survival signals, anti-apoptosis^18^, and synthesis of essential cellular proteins^19^. Numerous studies reported inflammation as an integral part of cerebral ischemia and therefore responsible for the poor outcome^20^. Modulation of immune cells in CNS disorder, especially in stroke, has been reported to be beneficial. Until now, the impact of GPR35 activation in cerebral ischemia is not known. Therefore, we investigated the role of GPR35 activation by pamoic acid in a mouse model of stroke. Our data reveal that activation of GPR35 by pamoic acid reprograms monocytes that results in improved stroke outcome.

## Results

### Pamoic acid mediates the neuroprotective effect of GPR35

**Figure 1:**
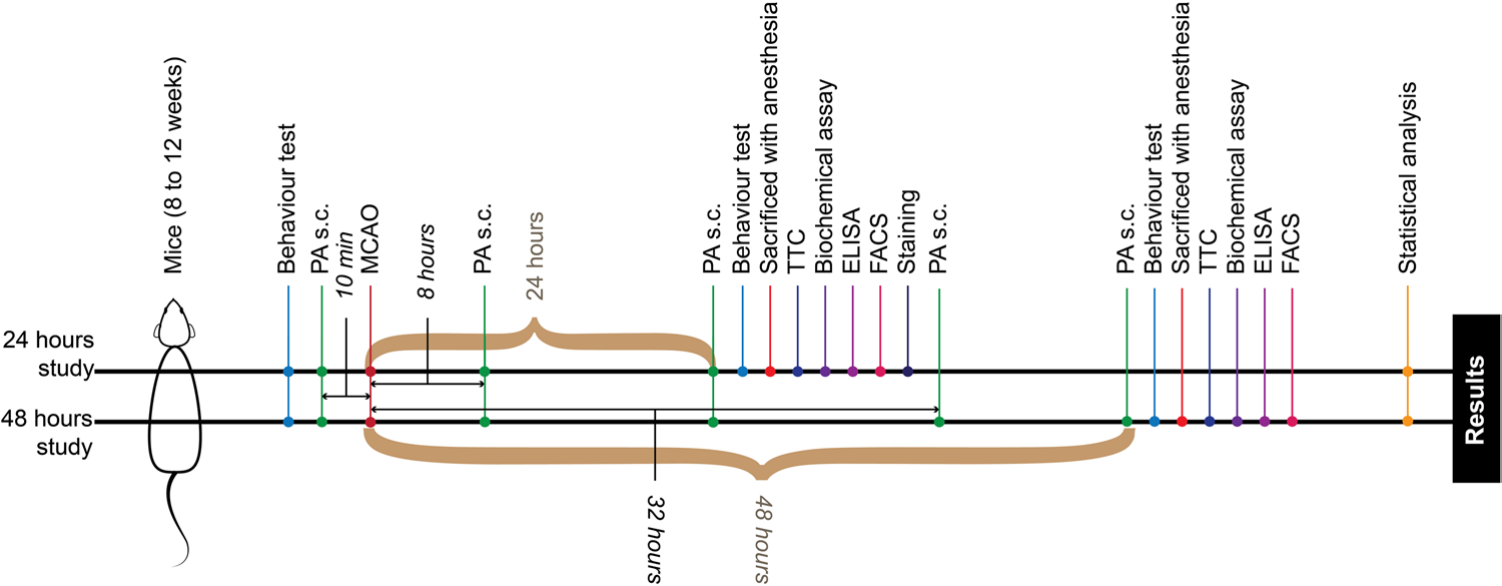
Experimental timeline. For the 24 h study, pamoic acid (PA) s.c. at a dose of 50 mg/kg and 100 mg/kg was administered 10 min before Middle Cerebral Artery Occlusion (MCAO) and 8 and 24 h after MCAO. For the 48 h study, pamoic acid (PA) s.c. at a dose of 100 mg/kg was administered 10 min before MCAO and 8 h, 24 h, 32 h, and 48 h after MCAO. ML194 s.c. or Triciribine i.p. was administered 20 min before MCAO and at 8 h and 24 h after the MCAO.

**Figure 2:**
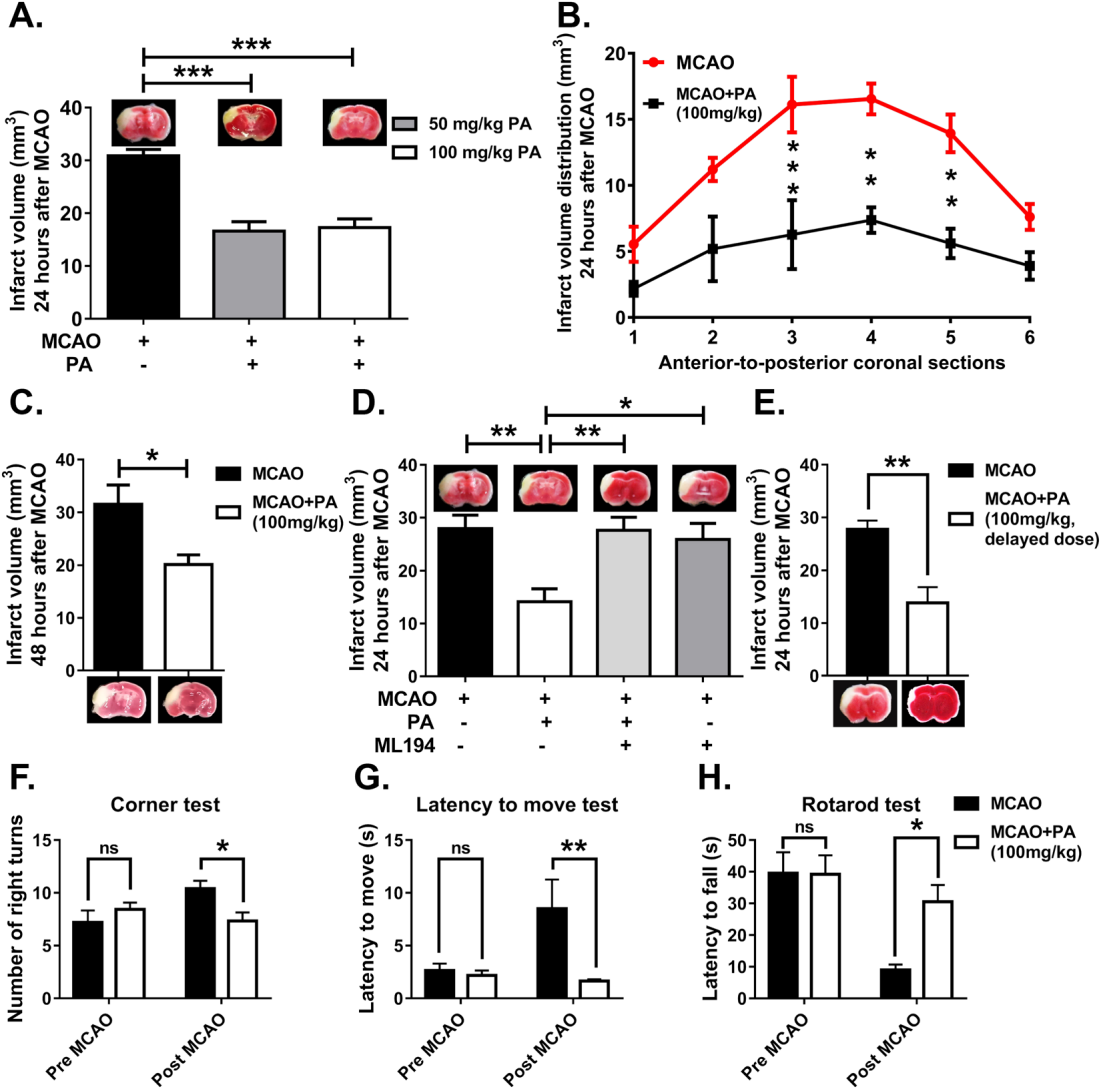
Pamoic acid (PA) prevents cerebral ischemia and improves sensorimotor dysfunction. A. PA treatment reduced infarct size 24 h after the MCAO at a dose of 50 mg/kg and 100 mg/kg body weight. One-Way ANOVA, F_(2/19)_ = 32.54, ***P = 0.001, (Bonferroni multiple comparison test) Values are means ± s.e.m, (n for MCAO = 10, n for 50 mg/kg PA = 7, and n for 100 mg/kg PA = 5). B. PA significantly affected the infarct distribution 24 h after the MCAO at a dose of 100 mg/kg. Two-Way ANOVA, F_(1/70)_ = 45.1,**P ≤ 0.0065,***P = 0.0008, (Bonferroni multiple comparison test) Values are means ± s.e.m, n = 4. C. PA is neuroprotective 48 h after the MCAO at a dose of 100 mg/kg BW. *P = 0.0136 (unpaired t-test), values are means ± s.e.m, (n for MCAO = 6, and n for MCAO + PA = 7). D. PA treatment (100 mg/kg) lost its activity in the presence of ML194 (0.7 mg/kg, s.c, 20 min before PA injections) 24 h after the MCAO. One-Way ANOVA, F_(3/24)_ = 6.957, *P = 0.0256, **P ≤ 0.0055, (Bonferroni multiple comparison test) Values are means ± s.e.m, n = 6–8, (n for MCAO = 7, and n for MCAO + PA = 8, MCAO + PA + ML194 = 7, and MCAO + ML194 = 6). E. Delayed treatment (The first dose was 1 h after the MCAO) with PA (100 mg/kg) reduced the infarct size 24 h after the MCAO. **P = 0.0038 (unpaired t-test), values are means ± s.e.m, (n = 5). F. PA treatment reduced the number of right turns in corner test 24 h after MCAO (100 mg/kg). Two-Way ANOVA, F_(1/17)_ = 0.9158, *P = 0.0339, (Bonferroni multiple comparison test) Values are means ± s.e.m, (n for MCAO = 10, n for MCAO + PA = 9). G. Mice treated with PA improved the latency to move one full body length 24 h after MCAO(100 mg/kg). Two-Way ANOVA, F_(1/19)_ = 7.082, **P = 0.0049, (Bonferroni multiple comparison test) Values are means ± s.e.m, (n for MCAO = 11, n for MCAO + PA = 10). H. In the rotarod test, PA treatment (100 mg/kg) reduced the latency to fall from the rod 24 h after the MCAO. Two-Way ANOVA, F_(1/10)_ = 4.463, ***P = 0.001, (Bonferroni multiple comparison test) Values are means ± s.e.m, (n for MCAO = 5, n for MCAO + PA = 6).

GPR35 is activated by pamoic acid^14^, which is currently used in many pharmaceutical preparations to modify dissolution rate^16^, and release property^21^. In our study (Fig. 1), We noticed pamoic acid treatment reduced the infarct size significantly at 100 mg/kg as well as 50 mg/kg body weight at 24 h (Fig. 2A,B) and 48 h (Fig. 2C) after the Middle Cerebral Artery Occlusion (MCAO). Since pamoic acid is a ligand for GPR35, we sought to investigate whether GPR35 mediates the neuroprotective effect of pamoic acid. Therefore, we repeated the experiment with pharmacological inhibition of the GPR35 using ML194^22,23^. We noticed that pamoic acid was only effective in the absence of ML194 (Fig. 2D), and the effect was lost in mice after MCAO that were treated with ML194. These data demonstrate that pamoic acid produces a neuroprotective effect by activating GPR35. Since the presentation of the stroke patient is often delayed in clinical settings, we sought to investigate whether pamoic acid could exert its neuroprotective effect when administered after the stroke incidence. We administered pamoic acid (100 mg/kg) one hour after the MCAO in a separate study and found that pamoic acid was still effective in reducing the infarct volume significantly (Fig. 2E).

Pamoic acid also improved the stroke-induced sensorimotor dysfunctions as evaluated by corner test, latency-to-move test, and rotarod test. In the corner test, bias towards the right side after MCAO was observed, which was normalized by pamoic acid treatment (Fig. 2F). After 24 h of MCAO, the latency-to-move one body length was increased significantly. However, mice that were treated with pamoic acid took significantly less time to perform the task (Fig. 2G). In the rotarod test, latency to fall from the rod was reduced after MCAO. However, pamoic acid treatment restored this parameter (Fig. 2H)

### Cellular expression of GPR35 in the ischemic brain

**Figure 3:**
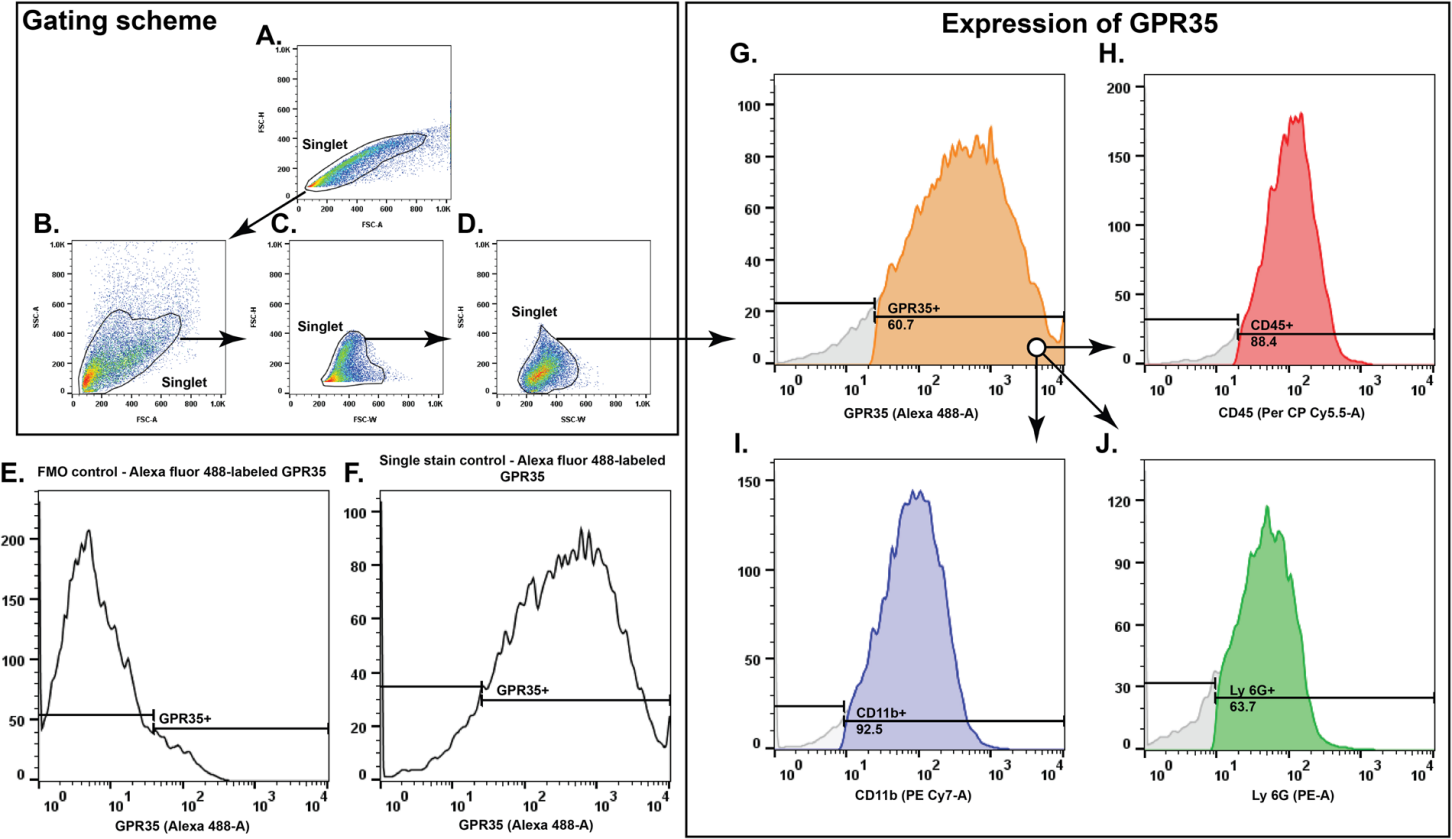
Cellular expression of GPR35 in the ischemic brain. (**A–D**) Represents the gating scheme for doublet discrimination and isolation of the singlet population. (**E**) Fluorescence minus one (FMO) control for Alexa fluor 488 labeled GPR35 positive cells. (**F**) Single stain control for Alexa fluor 488 labeled GPR35 positive cells. (**G–J**) Representation of the flowcytometry analysis revealing that the CD45 (**H**), CD11b (**I**), and Ly-6G (**J**) positive cells express GPR35 (**G**).

We performed flowcytometry on isolated brain cells and sorted out the singlets (Fig. 3A–D).

The expression of GPR35 in the ischemic brain was localized by staining the cells with the GPR35 antibody (Fig. 3E,F). We noticed that CD45, CD11b, and Ly-6G positive cells expressed GPR35 (Fig. 3G–J), indicating that GPR35 is expressed on monocytes/macrophages and neutrophils that might infiltrate the ischemic brain.

**Figure 4:**
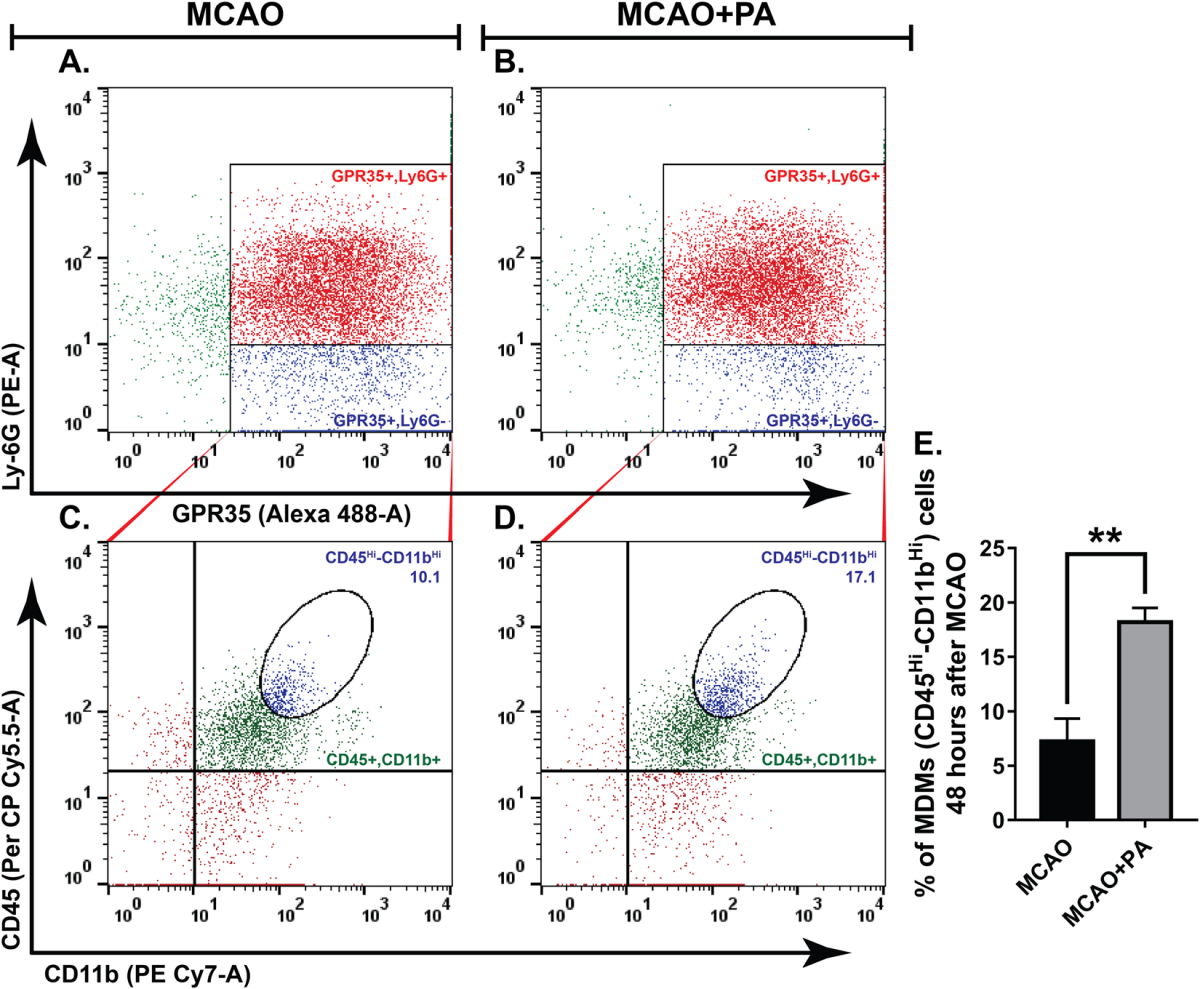
Pamoic acid (PA) treatment recruited monocyte-derived macrophages in the ischemic brain. (**A,B**) GPR35^+^- Ly-6G^**−**^ cells were gated and plotted against CD45 and CD11b (**C,D**). E. Monocyte-derived macrophages (MDMs) were identified as CD45^Hi^-CD11b^Hi^. The treatment with PA increased the GPR35^+^  MDMs 48 h after the MCAO. **P = 0.0044 (unpaired t-test), values are means ± s.e.m, n = 4.

Further analysis revealed that a substantial number of the gated GPR35^+^- Ly-6G^**−**^ cells were also positive for CD45^Hi^-CD11b^Hi^, indicating that these were monocyte-derived macrophages (MDMs)^24^ (Fig. 4A–D). Interestingly, pamoic acid treatment significantly increased the number of GPR35^+^ MDMs (Fig. 4E) 48 h after the MCAO. These findings suggest that the neuroprotective effect of pamoic acid is associated with an increased number of GPR35^+^   MDMs in a mouse model of stroke.

### Pamoic acid favors the infiltration of neuroprotective subsets of monocytes into the ischemic brain

**Figure 5:**
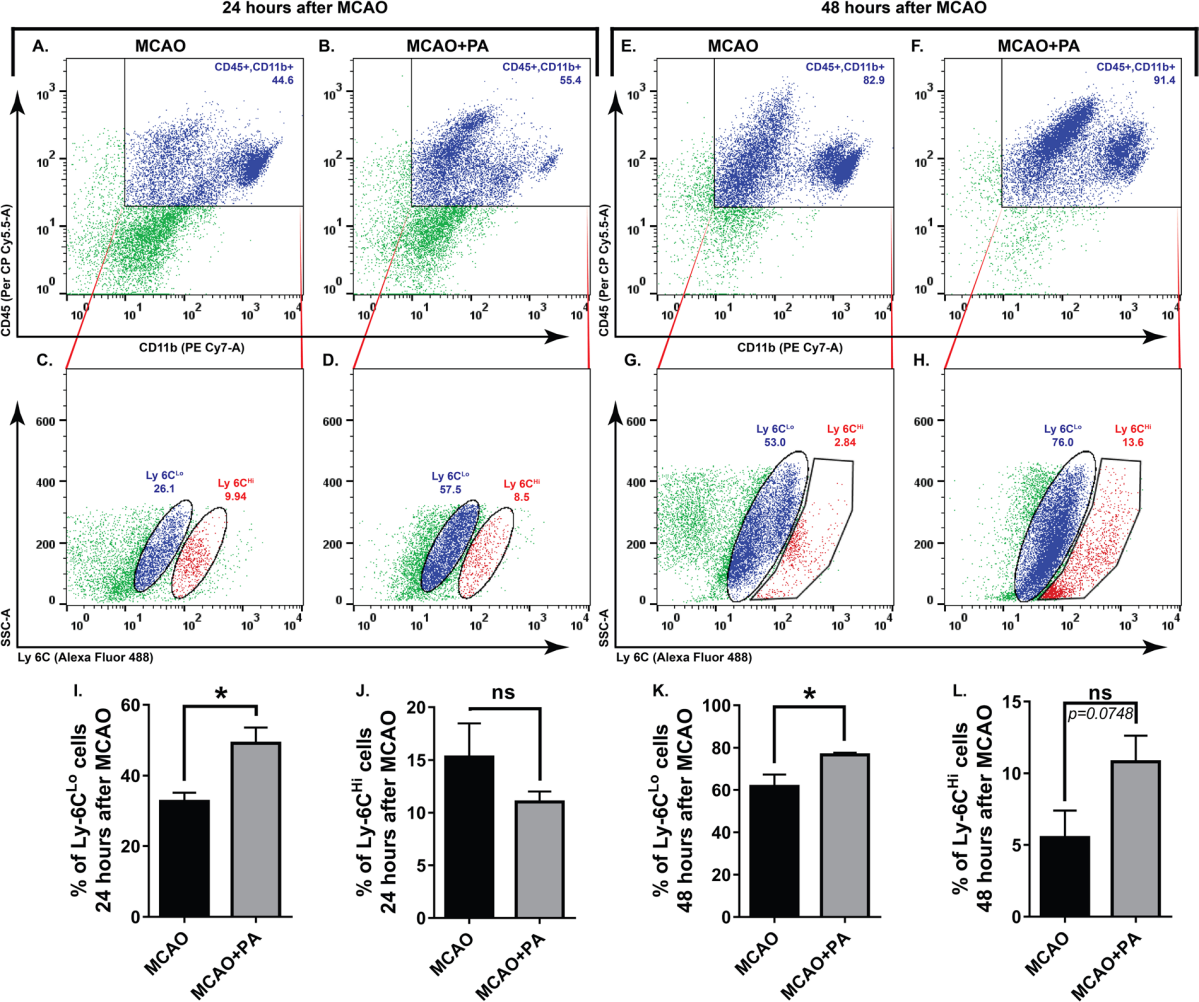
Characterization of infiltrated monocytes in the brain induced by pamoic acid (PA). The representative, flowcytometry diagram shows the Ly6C^Lo^ and Ly6C^Hi^ subsets of monocyte (**C,D,G,H**) gated from the CD45^+^ CD11b^+^ cell population (A, B, E, and F) in the ischemic hemisphere at both 24 h and 48 h after the MCAO respectively. I, J. PA treatment increased Ly-6C ^Lo^ cell population 24 h after the MCAO. *P = 0.0175, (unpaired t-test, n = 4). However, the Ly-6C ^Hi^ cell population was unaffected by PA treatment. K, L. PA treatment increased Ly-6C ^Lo^ cell population 48 h after the MCAO. *P = 0.0187, (unpaired t-test, n for MCAO = 5, n for MCAO + PA = 6). Ly-6C ^Hi^ cell population was unaffected by PA treatment at this time point.

**Figure 6:**
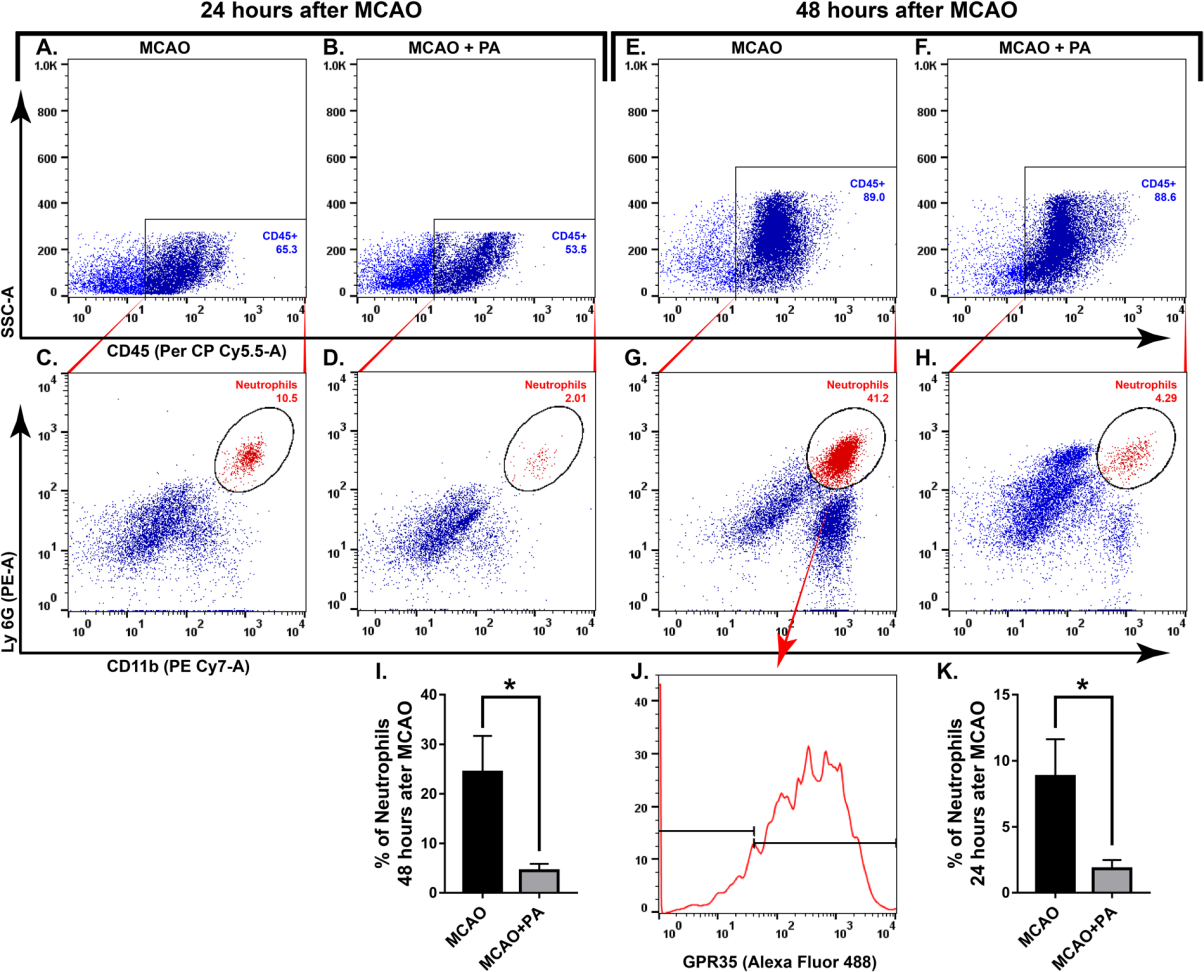
Pamoic acid (PA) treatment reduced the infiltration of GPR35 expressing neutrophil in the ischemic brain. The representative flow cytometry diagram shows the CD45 gated (**A,B,E,F**) Ly6G^Hi^ CD11b^Hi^ cells (**C,D,G,H**) in the ischemic hemisphere at both 24 h and 48 h after the MCAO respectively. These neutrophils (Ly6G^Hi^-CD11b^Hi^) also expressed GPR35 (J). I. PA treatment reduced infiltrating neutrophils 24 h after the MCAO. *P = 0.0408, (unpaired t-test), values are means ± s.e.m, n = 5. K. PA treatment reduced infiltrating neutrophils 48 h after the MCAO. *P = 0.0277, (unpaired t-test), values are means ± s.e.m, n = 5.

Since the number of MDMs that express GPR35 were increased after pamoic acid treatment in stroke, we further evaluated the impact of pamoic acid on monocyte infiltration into the ischemic brain in a separate cohort of study. We noticed that pamoic acid treatment preferentially increased the number of Ly6C^lo^ subset of monocyte in the ischemic hemisphere at both 24 h (Fig. 5A–D,I,J) and 48 h (Fig. 5E–H,K,L) after the MCAO. In contrast to monocytes, pamoic acid treatment significantly reduced the number of infiltrating neutrophils in the ischemic brain that express GPR35 both 24 h and 48 h after the MCAO (Fig. 6A–K).

### Akt and p38 MAPK signaling pathway mediates the neuroprotective effect of pamoic acid

**Figure 7:**
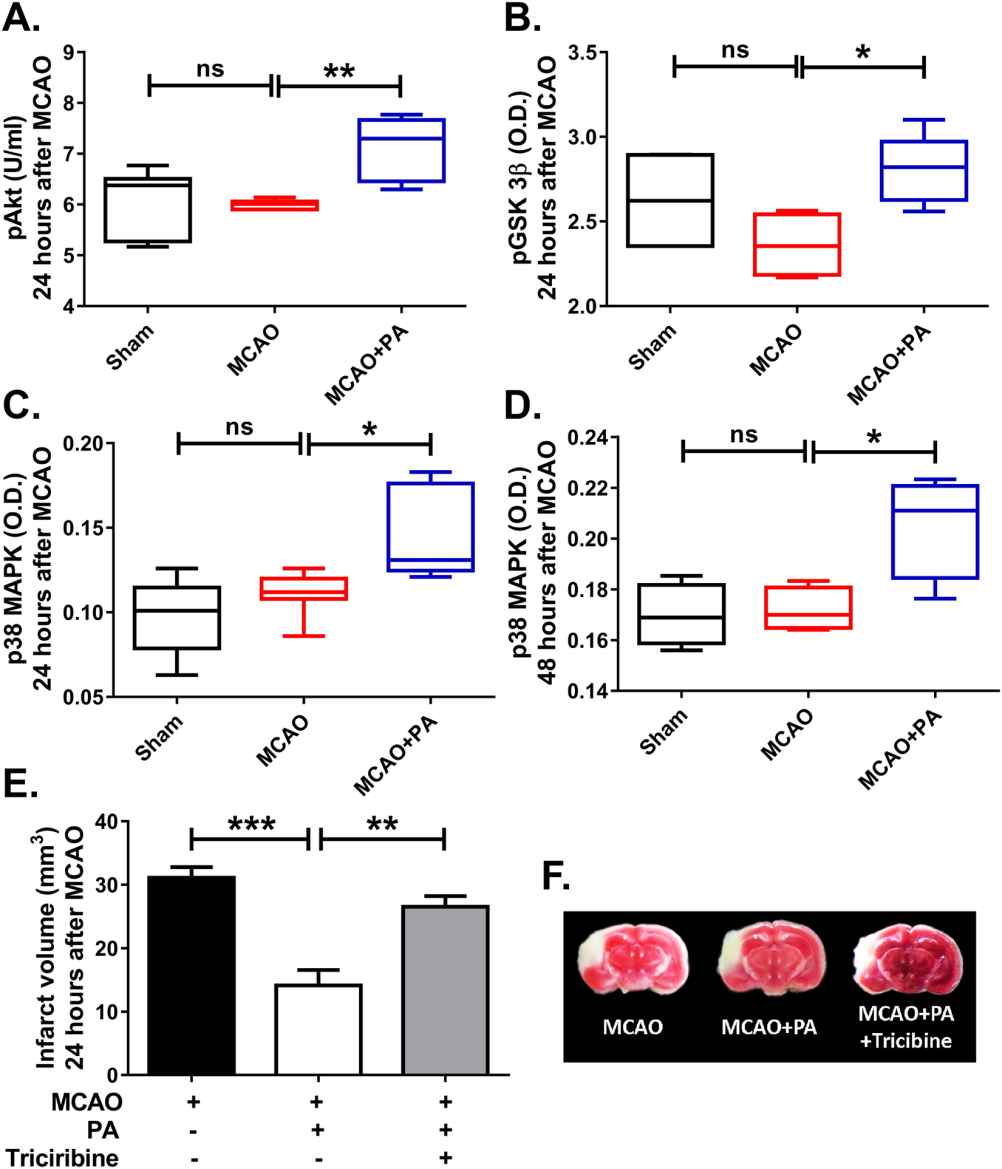
Pamoic acid (PA) activates Akt and p38 MAPK signaling in the ischemic brain. (**A**) PA treatment increased the concentration of pAkt at 24 hours after the MCAO, One-Way ANOVA, F_(2/18)_ = 9.515, **P = 0.0039 (Bonferroni multiple comparison tests), values are min to max. n = 7. (**B**) PA treatment increases the pGSK-3β at 24 h after the MCAO, one-Way ANOVA, F_(2/11)_ = 4.652, *p = 0.0332 (Bonferroni multiple comparison test) values are min to max, (n for Sham = 4, n for MCAO = 4, n for MCAO + PA = 6), (**C**) PA treatment increased the p38 MAPK at 24 h after the MCAO, One-Way ANOVA, F_(2/19)_ = 9.089, *P = 0.0221 (Bonferroni multiple comparison test), values are min to max, (n for Sham = 4, n for MCAO = 4, n for MCAO + PA = 6). (**D**) PA treatment increased the activity of p38 MAPK 48 h after the MCAO, One-Way ANOVA, F_(2/9)_ = 7.598, *P = 0.0290 (Bonferroni multiple comparison tests), values are min to max, n = 4. (**E,F**) The effect of PA was lost when combining with Triciribine, One-Way ANOVA, F_(2/17)_ = 19.21, **P = 0.0029, ***P = 0.0001 (Bonferroni multiple comparison tests), values are means ± s.e.m, (n for MCAO = 7, n for MCAO + PA = 8, MCAO + PA + Triciribine =5).

Akt and p38 MAPK signaling play an important role in human monocyte/macrophage survival^25^. These signaling pathways are reported to be critically involved in determining stroke outcomes^12,13^. GPR35 is known to activate p38 MAPK^26^. p38 MAPK is also known to mediate the chemoattractant function of CxCL17 (an endogenous ligand for GPR35), especially on monocyte and macrophages^27^. Considering such, we investigated the role of Akt and p38 MAPK in GPR35 mediated neuroprotection. We noticed that pamoic acid treatment significantly increased the concentration of phospho-Akt 24 h after the MCAO (Fig. 7A) in the ischemic hemisphere. Accordingly, the GSK3β activity was reduced upon pamoic acid treatment (Fig. 7B). However, 48 h after MCAO, the phospho-Akt levels were not different among the groups. Nevertheless, GSK3β phosphorylation was found to be increased at this time point (Supplementary Fig. 1A,B). When we measured the phospho-p38 MAPK level in the ischemic brain, we noticed that pamoic acid significantly increased the phospho-p38 MAPK level both at 24 h (Fig. 7C) and 48 h after the MCAO (Fig. 7D). To further delineate the role of Akt in pamoic acid mediated neuroprotection we treated the mice with Triciribine, an inhibitor of Akt that is known to inhibit the cellular activation of Akt1/2/3 selectively. We noticed that the neuroprotective effect of pamoic acid was abrogated when combining with Triciribine (Fig. 7E,F) 24 h after the MCAO. These observations indicate that pamoic acid mediated GPR35 activation is neuroprotective via Akt and p38 MAPK signaling pathways.

### Pamoic acid treatment ameliorates oxidative stress and iron deposition after stroke in mice

**Figure 8:**
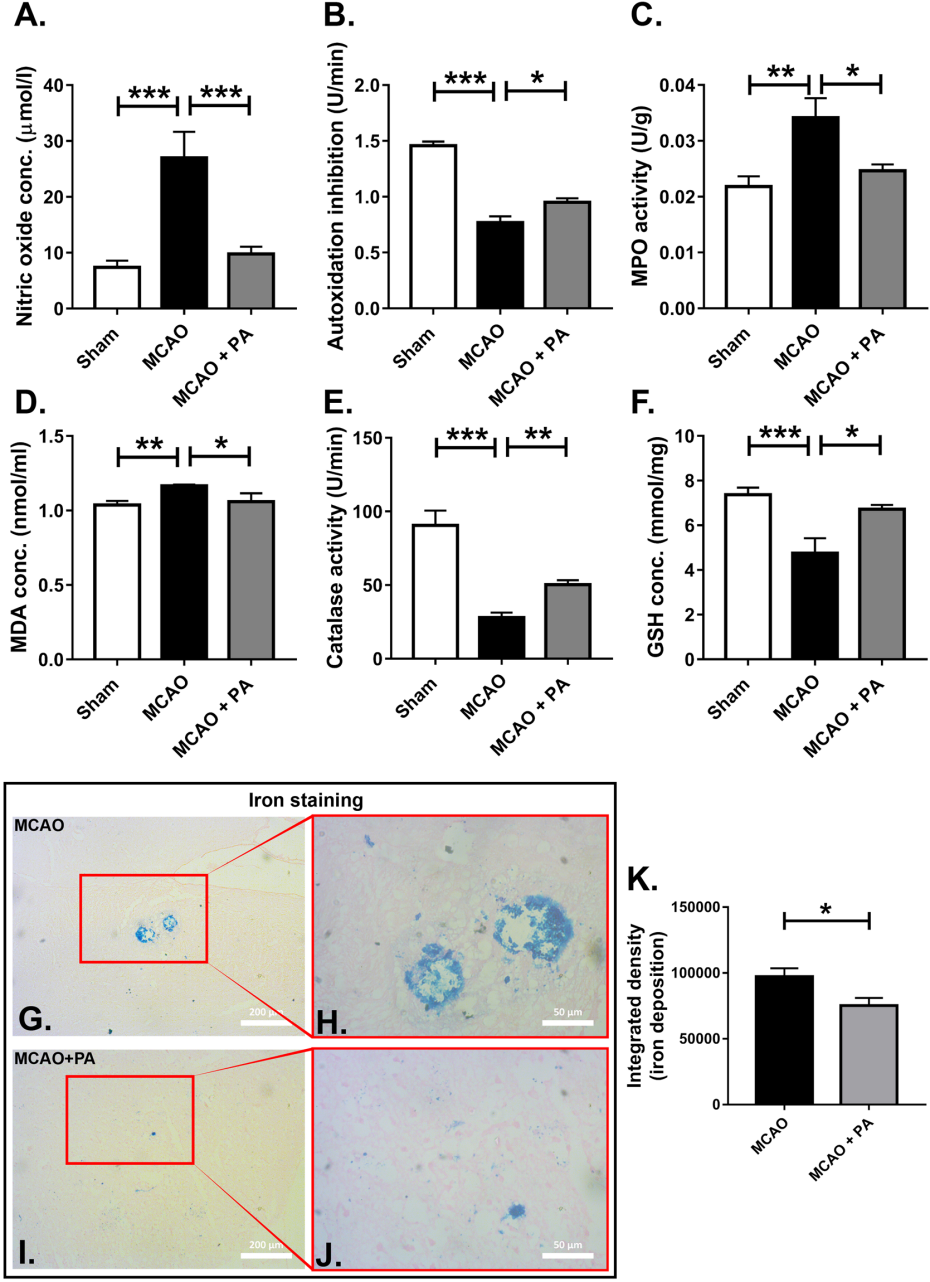
Pamoic acid (PA) reduces the oxidative stress and iron deposition in brain 24 h after MCAO. (**A**) PA reduced the nitric oxide concentration in the ischemic hemisphere 24 h after the MCAO. The One-Way ANOVA, F_(2/20)_ = 15.23, ***P = 0.0002–0.0007 (Bonferroni multiple comparison tests), values are means ± s.e.m, (n for Sham = 8, n for MCAO = 7, MCAO + PA = 8). (**B**) PA treatment increased autoxidation inhibition in the ischemic hemisphere 24 h after the MCAO, one-Way ANOVA, F_(2/25)_ = 67.55, *P = 0.0174, ***P = 0.0001 (Bonferroni multiple comparison test), values are means ± s.e.m, (n for Sham = 9, n for MCAO = 9, MCAO + PA = 10). (**C**) PA treatment reduced myeloperoxidase (MPO) activity in the ischemic hemisphere 24 h after the MCAO. The One-Way ANOVA, F_(2/16)_ = 7.772, *P = 0.0362, **P = 0.0046 (Bonferroni multiple comparison test), values are means ± s.e.m, (n for Sham = 7, n for MCAO = 6, MCAO + PA = 6). (**D**) PA treatment reduced malondialdehyde (MDA) concentration in the ischemic hemisphere 24 h after the MCAO. The One-Way ANOVA, F_(2/22)_ = 7.285, *P = 0.0384, **P = 0.0066 (Bonferroni multiple comparison test), values are means ± s.e.m, (n for Sham = 7, n for MCAO = 11, MCAO + PA = 6). (**E**) PA treatment increased the catalase activity in the ischemic hemisphere 24 h after the MCAO. The One-Way ANOVA, F_(2/20)_ = 34.72, **P = 0.0057, ***P = 0.0001 (Bonferroni multiple comparison test), values are means ± s.e.m, (n for Sham = 5, n for MCAO = 8, MCAO + PA = 10). (**F**) PA treatment increased the GSH activity in the ischemic hemisphere 24 h after the MCAO. The One-Way ANOVA, F_(2/22)_ = 10.93, *P = 0.0102, ***P = 0.0004 (Bonferroni multiple comparison test), values are means ± s.e.m, (n for Sham = 10, n for MCAO = 7, MCAO + PA = 8). (**G,H,I,J**) Iron deposition in the ischemic hemisphere 24 h after the MCAO. (**K**) PA treatment reduced the integrated density 24 h after the MCAO. *P = 0.0248 (unpaired t-test), values are means ± s.e.m, (n for MCAO = 8, MCAO + PA = 7).

Oxidative stress is critically associated with ischemic brain damage. Nitric oxide (NO) is an inorganic gas and known to play a significant role in modulating neuronal activity^28^. However, under ischemic conditions, it is generated in excess quantities and intervenes in inflammatory and cytotoxic action leading to neuronal death and poor outcome in stroke^29^. In line with others, we noticed an increase in NO release as measured by nitrate concentrations after ischemia in the brain of mice, and pamoic acid normalized the NO concentration 24 h and 48 h after the MCAO (Fig. 8A and Supplementary Fig. 2A). Superoxide dismutase (SOD), catalase, and reduced glutathione (GSH) are the natural antioxidants that neutralize free radicals. When we measured SOD, catalases and GSH in brain tissue, we found that the concentrations of SOD (Fig. 8B and Supplementary Fig. 2B), catalase (Fig. 8E and Supplementary Fig. 2D), and GSH (Fig. 8F and Supplementary Fig. 2E) increased substantially after stroke with pamoic acid treatment. Neutrophils and monocytes are the known source of myeloperoxidase (MPO)^30^. Increased MPO activity was observed in the brain after MCAO, which was normalized when mice were treated with pamoic acid (Fig. 8C and Supplementary Fig. 2C). Malondialdehyde (MDA) is a marker of lipid peroxidation associated with oxidative stress. In our study, pamoic acid treatment significantly reduced the MDA concentration after 24 h of stroke compared to the saline-treated mice (Fig. 8D and Supplementary Fig. 2F).

Iron homeostasis is crucially involved in normal brain function. Iron overload during ischemic injury may induce oxidative stress leading to apoptosis. Under ischemic conditions, superoxide and NO are known to facilitate the process of ferritin-bound iron release substantially^31,32^. In our current study, we noticed increased iron deposition after stroke compared to the pamoic acid treated mice (Fig. 8G–K).

## Discussion

Our study revealed the neuroprotective effects of pamoic acid in a mouse model of stroke. Pamoic acid reduced the infarct volume, most likely in a GPR35 dependent manner (Fig. 2). Pamoic acid treatment activated neuroprotective subsets of monocytes/macrophages that resulted in improved functional outcome after cerebral ischemia. Till to date, the only FDA approved pharmacotherapy for stroke is tissue plasminogen activator (tPA). However, this therapy is limited to a golden timeframe of 4 hours of stroke incidence. Pamoic acid in this context showed promising effect in our current study. It reduced the infarct volume substantially when administered in a delayed time point after the stroke incidence (Fig. 2E).

Previous studies showed that GPR35 is expressed in CA1 neurons apart from immune cells and involved in the control of neuronal activity in the hippocampus^6^. Activation of GPR35 by pamoic acid, kynurenine, and zaprinast is associated with antinociceptive activity^14,33^. Pamoic acid, on the other hand, is known to increase the phosphorylation of extracellular signal-regulated kinase 1/2 in a G_i/o_-linked GPR35 dependent manner^14^. A separate study reported that GPR35 expressed in adipose tissue increased energy expenditure and is involved in the regulation of inflammation by enhancing the expression of anti-inflammatory genes^34^. So far, the potential of GPR35 to improve CNS disorders especially stroke was unknown. Here we provide evidence that in an ischemic condition, neutrophils and monocyte/macrophages that infiltrated the ischemic brain through the leaky blood-brain barrier (BBB) expressed GPR35. Treatment with pamoic acid increased the number of GPR35 expressing MDMs in the ischemic brain and improved stroke outcomes. Pamoic acid treatment also reduced the number of neutrophils and associated MPO activity in the ischemic brain.

Neuroinflammation plays a critical role in the pathogenesis of ischemic stroke. It may lead to both beneficial and detrimental consequences^35,36^. Due to compromised BBB function, blood-borne immune cells infiltrate into the ischemic brain during a stroke. Neutrophils are the first cell type to appear at the ischemic brain, followed by monocytes and macrophages. Although depletion of neutrophil resulted in neuroprotection across several studies, monocytes/macrophage ablation turned out to be either detrimental or did not benefit stroke outcomes^3,37^. MDMs that infiltrate the ischemic brain in a CCR2-dependent manner exhibit a high degree of functional plasticity and contribute to the post-ischemic repair mechanisms^38,39^. In line with other elegant studies^24^, we noticed MDMs in the ischemic hemisphere, and the number of these cell types were augmented in the ischemic hemisphere when treated with the GPR35 agonist pamoic acid (Fig. 4). Interestingly, pamoic acid treatment reduced the number of infiltrating neutrophils in the ischemic hemisphere in contrast to its effect on MDMs (Fig. 6).

Circulating monocytes exhibit two distinct phenotypes with inimitable functional properties. In mice, monocytes that express a high level of Ly-6C (Ly-6C^Hi^) are known as the classically activated pro-inflammatory subset, which is specifically recruited to injury sites. On the other hand, monocytes with a low level of Ly-6C (Ly-6C^Lo^) expression are considered to be anti-inflammatory and involved in repair mechanisms after injury^40,41^. In contrast to this traditional concept of phenotypes, a growing body of evidence suggest that while recruited into the inflamed tissue, Ly-6C^Hi^ monocytes may differentiate into both classically activated pro-inflammatory macrophages (M1) and alternatively activated (M2) anti-inflammatory macrophages. Unlike Ly-6C^Hi^ monocytes, Ly-6C^Lo^ expressing monocytes that are recruited during inflammation may only differentiate into M2 macrophages^42^. The current consensus suggests that during a stroke, peripheral monocytes/macrophages that are recruited early (Ly-6C^Hi^) into the ischemic brain, becoming M1 tissue macrophages. Afterward, these cells lose their Ly-6C and CCR2 expression and become capable of releasing repair mediators^43,44^. In our current study, we found that the number of Ly-6C^Lo^ expressing monocytes was increased significantly in the ischemic hemisphere upon pamoic acid treatment 24 h and 48 h after the MCAO (Fig. 5). After 48 h of MCAO, we noticed a slight increment in Ly-6C^Hi^ expressing monocytes upon pamoic acid treatment, which may reflect the dynamics between the protective monocytes subset (Ly-6C^Lo^) and the inflammatory Ly-6C^Hi^ expressing monocytes^45^.

Oxidative stress plays a critical role in reconciling ischemic brain damage. Cells maintain a low concentration of reactive oxygen species (ROS) to perform various functions^46^. However, to titrate the excess ROS produced during a stroke, endogenous antioxidants such as SOD and catalase are crucial. It is especially critical for the brain not only because neurons express a low level of antioxidant enzymes and have a high basal oxygen consumption rate, but also because concentrations of oxidizable lipids and iron that can act as pro-oxidant are very high^47,48^. In this context, p38 MAPK is of special importance. Apart from playing a crucial role in the monocyte differentiation and chemotaxis^49^, it substantially reduces the ROS mediated brain damage by enhancing the expression of SOD and catalase^50^. It is also implicated in the survival of endothelial cells in cerebral ischemia^51^. Cardiac ischemia is known to be improved upon p38 MAPK activation by carbon monoxide^52^. In line with these observations, we noticed that pamoic acid treatment enhanced the activity of antioxidant enzymes SOD, catalase, and GSH (Fig. 8) and reduced iron overload (Fig. 8G–K). Accordingly, it also increased the phosphorylation of p38 MAPK (Fig. 7C,D).

The PI3K/Akt pathway is known to regulate the survival, migration, and proliferation of macrophages and coordinate their response to diverse metabolic and inflammatory stimuli^18^. Akt activation is deemed necessary to facilitate M2 polarization of macrophages since its inhibition results in the abrogation of M2 gene expression^53^. Furthermore, signals such as BMP-7 and TGFβ promote M2 polarization through PI3K/Akt signaling^54,55^. Inhibition of PI3K/Akt signaling in synovial macrophages from rheumatoid arthritis patients is associated with increased apoptotic cell death^56^. Akt activation in macrophages results in reduced severity in experimental autoimmune encephalomyelitis^57^. Akt signaling was implicated in neuroprotection after stroke^13^. A recent study demonstrated that in non-small-cell lung cancer, Akt inhibition resulted in suppression of GPR35 expression^58^. In our study, we found that pamoic acid mediated neuroprotection is associated with increased phosphorylation of Akt, and pharmacological inhibition of Akt phosphorylation resulted in the abrogation of neuroprotection in stroke (Fig. 7A,E,F). GSK3β is a downstream signaling molecule of the PI3K/Akt cascade. It is known to be negatively regulated by Akt and its inhibition is associated with improved cognitive function after stroke^59^. p38 MAPK on the other hand, may also inactivate GSK3β in the brain by direct phosphorylation at its C terminus leading to beta-catenin accumulation and thus providing a p38 MAPK-mediated survival signal^60^. Indeed, in our study, we noticed increased phosphorylation of GSK3β upon pamoic acid treatment at both the time point after MCAO (Fig. 7B and Supplementary Fig. 1B) although the pAkt concentration was unaffected 48 h after the MCAO. Therefore, we conclude that activation of GPR35 on monocyte/macrophages by its ligand pamoic acid reprograms these cell types into neuroprotective pathways.

## Method

### Mice

Male Swiss albino mice (8–12 weeks) were collected from the North South University (NSU) animal house and were maintained under standard environmental conditions (temperature 23.0 ± 2.0 °C, relative humidity: 55–65% and 12 h light and dark cycle). All experiments were carried out according to the institutional guideline and were approved by the NSU Institutional Animal Care and Use Committee (IACUC).

### Mouse stroke model

In this model, mice were subjected to left middle cerebral artery occlusion (MCAO) as described previously^3^. The mice were anesthetized with 2.5% 2,2,2-tribromoethanol (15 µl/g BW, i.p., CAS 75-80-9, Sigma-Aldrich). The skin between the ear and the orbit on the left side was incised, and the temporal muscle was removed. The stem of the middle cerebral artery (MCA) was exposed by drilling a burr hole and occluded using microbipolar electrocoagulation. The surgery was performed under a stereomicroscope, and the body temperature was maintained using a heating pad. The skin incision was closed, and mice were placed under the heating lamp until full recovery. After 24 h or 48 h of MCAO, mice were reanesthetized, and intracardiac perfusion with saline was performed. After removal, brains were placed in a brain matrice to obtain 1 mm thick coronal sections and were stained with 2,3,5-triphenyl-tetrazolium chloride solution (CAS 298-96-4; Loba Chemie)^61^. The stained sections were digitalized, and the infarct volume was determined using ImageJ. The calculated infarct volume was corrected for brain edema as described previously^62,63^. Mice were randomized to treatment groups. Pamoic acid (PA, 100 mg/kg BW, 50 mg/kg BW, s.c.; CAS: 130-85-8, Sigma-Aldrich)^64^ or vehicle was administered subcutaneously 10 min before MCAO and 8 h, 24 h, 32 h, and 48 h after MCAO if not mentioned otherwise. ML194 (0.7 mg/kg BW, s.c., CID 9581011, Sigma-Aldrich) and Triciribine (i.p. 1 mg /kg BW, CAS: 35943-35-2, Sigma-Aldrich) were injected 20 min before MCAO and eight hours, 24 h, 32 h, 48 h after MCAO if not mentioned otherwise. The investigators were blinded to the treatment groups. The experimental timeline has been illustrated in Fig. 1.

### Behavioral analysis

To evaluate the sensorimotor function, we used the corner test, latency–to-move test, cylinder test, and rotarod test. These tests have been described previously^62^. In the corner test, mice were placed to enter into a 30°corner before and 24 hours after MCAO. When the mice reached the corner, they turned either left or right on rearing. The number of rights and left turns was counted out of 12 trials.

The latency-to-move test was performed by placing the mice at the center of a plain board. The time to cross one body length was measured before and 24 hours after MCAO.

Motor coordination and balance alterations of the mice were evaluated using a rotarod test^65^. During a four day training before surgery, mice were placed on the rotarod for the 30 s with no rotation, then 1 min with a rotation of 8 rpm. Mice were placed on the rod until they were able to stay for 1 min. During the test session, the latency-to-fall was recorded for each mouse to compare motor coordination.

### Flowcytometry

Flowcytometry was performed as described previously^66^. Mice were anesthetized 24 or 48 h after MCAO and perfused intracardially with saline. Brains were harvested, and the left hemispheres were digested for 30 min at 37 °C in digestion media containing DMEM (Invitrogen), collagenase A (1 mg/ml, Roche), and DNAse (0.1 mg/ml, Roche). The cells were filtered through a 40 µm cell strainer. Percoll gradient (GE Healthcare, 78%, and 30%) was used to separate myelin and debris. The cells were collected from the interface of the gradient and washed with PBS. After treatment with rat anti-mouse CD16/32 (Invitrogen, 1:100) for 10 min, the cells were incubated with antibodies for 30 min as follows: PerCP-labeled rat anti-mouse CD45 (Invitrogen, 1:100), PE-Cy7-labeled rat anti-mouse CD11b (Invitrogen, 1:100), PE-labeled Ly-6G (Invitrogen, 1:100), and Alexa 488-labeled Ly-6C (Invitrogen, 1:100). GPR35 was labeled with rabbit anti mouse GPR35 polyclonal primary antibody (1:100, Invitrogen, Catalog # PA5-23237) and Alexa Fluor 488-labeled goat anti-rabbit secondary Antibody (1: 100, Invitrogen, Catalog # A-11034). The cells were analyzed on BD fusion (BD Bioscience, 100 µm nozzle) with the laser 488 nm.

### Protein estimation

Protein concentration was estimated from the brain samples as described previously^67^. Briefly, a 20 µl sample was taken in a 1.5 ml tube. 20 µl of sodium hydroxide solution was added and heated at 100 °C in a water bath for 10 min. The samples were then allowed to cool in room temperature, and 200 µl complex reagent was added with the sample mixture and incubated for 10 min. 20 µl Folin reagent was added and incubated for 60 min. The samples were then read at 750 nm using a microplate reader.

### MDA (Malondialdehyde) assay

Based on protein estimation, 8 µl to 15 µl of the samples were taken in a 1.5 ml tube. To this, 250 µl trichloroacetic acid was added. To this, a 283–295 µl of distilled water was added based on protein estimation. The mixture was centrifuged for 20 min, and a 500 µl supernatant solution was collected. To this, 500 µl thiobarbituric acid was added and incubated in the dark for 15 hours. The absorbance of the mixture was measured at 630 nm using a microplate reader and concentration was determined using a standard curve^68^.

### Superoxide dismutase (SOD) assay

Superoxide dismutase activity was measured as described previously^69^. Briefly, auto-oxidation of epinephrine at pH 10.4 was spectrophotometrically measured. 8 µl to 15 µl of the samples were added with 0.02 ml epinephrine. After 5 min, the absorbance was measured at 490 nm. The activity of SOD was expressed as a unit of autooxidation inhibition per minute.

### Catalase (CAT) assay

Catalase concentration was measured as described earlier with slight modifications^70^. Based on protein estimation, 8 µl to 15 µl of samples were taken in a 96 well plate. To this 50 µl of hydrogen peroxide and 84 µl to 92.5 µl of PBS was added. The changes in absorbance of the reaction solution at 405 nm were then determined at 0 seconds, 30 seconds, 60 seconds, 120 seconds, 3 minutes, and 4 min. One unit of catalase activity was defined as an absorbance change of 0.01 as units/min.

### NO assay

NO concentration was measured as described earlier with modification^71^. Briefly, an equal volume of sample and Greiss reagent were mixed and incubated for 10 min at room temperature in the dark. The absorbance was determined at 450 nm. The concentration of nitrate was measured using a standard curve.

### MPO (Myeloperoxidase) assay

MPO activity was measured as described previously^72^ with modifications. Briefly, 8 µl to 15 µl of samples were taken in a 96-well plate. To this, equal amounts of hydrogen peroxide and O-Dianisidine were added. The absorbance was measured at 450 nm in a spectrophotometer. The activity was measured from the change in absorbance over 1 min period.

#### **Histological assay**

The iron staining was performed using a Prussian blue reaction with a slight modification of Mallory’s method^73^. The formalin-fixed brain tissue was paraffinized, and 5 µm thick sections were obtained using a microtome. After deparaffinization, the sections were treated with different concentrations of ethanol for 5 min. The slides were then transferred to a mixture of 5% potassium ferrocyanide and 5% hydrochloric acid solution for 5 min. The slides were washed under tap water and treated with eosin for 2 min. Finally, the slides were dehydrated with different concentrations of ethanol and mounted.

## ELISA

### pAkt

The enzyme-linked immunosorbent assay (ELISA) of pAkt was performed using the AKT (Phospho) [pT308] sandwich ELISA Kit (Invitrogen, Catalog No KHO0201) according to manufacture’s protocol. The AKT [pT308] standard dilution was prepared by reconstituting the standard with 100 Units/mL Standard dilution buffer, and serial dilution was performed to achieve 7 different concentrations of 50, 25, 12.5, 6.25, 3.12, 1.6 and 0 Units/mL AKT [pT308]. The absorbance assay of the standard dilution was used to construct the standard curve.

### p38 MAPK and GSK 3β

The ELISA of p38 MAPK and GSK 3β was performed using the InstantOne p38 MAPK ELISA kit (Invitrogen, ref. no. 85-86023-11) and InstantOne GSK 3β ELISA kit (Invitrogen, ref. no. 8586173-11). The assay was conducted according to the instruction manual of the manufacturer.

### Statistical analysis

Values in the manuscript are expressed as mean ± s.e.m. Two groups were compared using the t-test. For comparing more than two groups, one-way analysis of variance (ANOVA) followed by Bonferroni’s post hoc or two-way ANOVA followed by Bonferroni’s post hoc test were employed.

## Supplementary information

Supplemental information.
