# Supplementary material for: Activation of GPR35 protects against cerebral ischemia by recruiting monocyte-derived macrophages
Source: Sci Rep. 2020 Jun 10;10:9400. doi: 10.1038/s41598-020-66417-8 (PMC7287103; doi:10.1038/s41598-020-66417-8)
Supplement: Supplementary file 1 — Supplemental information. [file 41598_2020_66417_MOESM1_ESM.pdf]

Supplementary information

Activation of GPR35 protects against cerebral ischemia by recruiting monocyte-derived macrophages

Ozayra Sharmin[1,+], Ariful Haque Abir[1,+], Abdullah Potol[1,2], Mahabub Alam[1], Jewel Banik[1,3], A.F.M. Towheedur Rahman[1,4], Nuzhat Tarannum[1], Rasiqh Wadud[1,5], Zaki Farhad Habib[1,6], and Dr. Md. Mahbubur Rahman[1,*]

[1]Laboratory of Pharmacology, Department of Pharmaceutical Sciences, North South University, Bashundhra R/A, Dhaka-1229, Bangladesh

Current affiliations:

[2]Faculty of Medicine, Friedrich Schiller University Jena, 07743 Jena, Germany

[3]Deptartment of Neurobiology & Developmental Sciences, College of Medicine, UAMS, 4301 W. Markham St., Little Rock, AR 72205, USA

[4]Milwaukee Institute of Drug Discovery, Department of chemistry and Biochemistry, University of Wisconsin-Milwaukee, WI 53211, USA

[5]Department of Veterinary Medicine, University of Cambridge, Madingley Road, Cambridge CB3 0ES, UK

[6]Department of Physiology, Development and Neuroscience, University of Cambridge, Downing Street, Cambridge, UK

[*]Author of correspondence

Dr. Md. Mahbubur Rahman

SAC1059, Department of Pharmaceutical Sciences,

North South University, Bashundhara R/A, Dhaka-1229, Bangladesh.

Email: rahman.mahbubur@northsouth.edu

[+]These authors contributed equally

Results

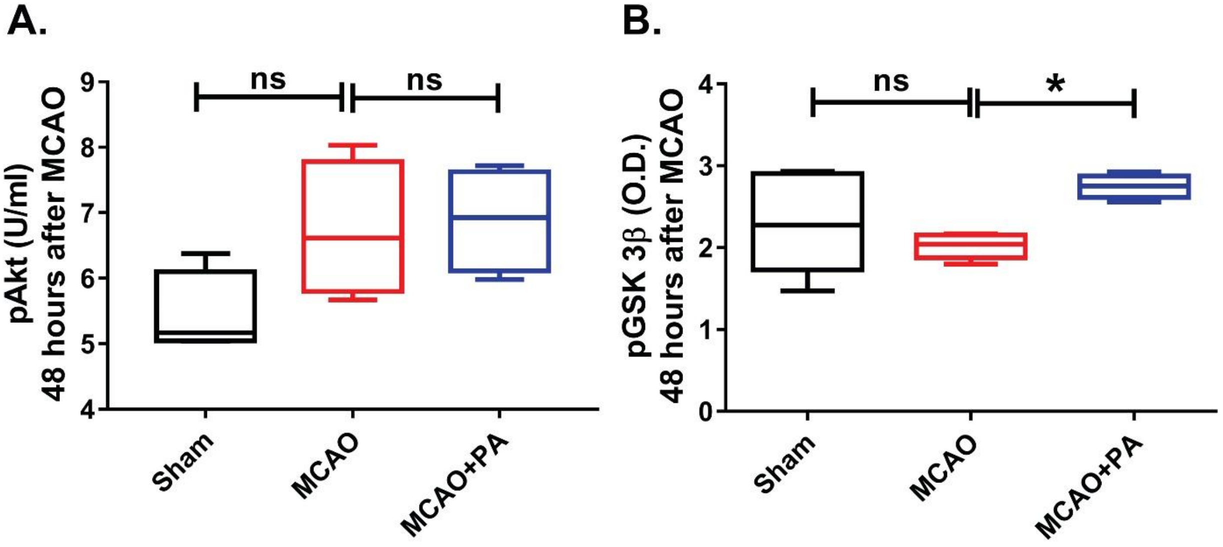

Figure 1: Effect of PA on pAkt and pGSK-3β 48 h after the MCAO.

A. Phospho-Akt concentration was unaffected in the ischemic brain upon pamoic acid treatment at 48 h after the MCAO The One-Way ANOVA, $P>0.05$ (Bonferroni multiple comparison test), values are means±s.e.m, n=4. B. PA treatment significantly increased the phosphorylation of GSK-3β after 48 h of MCAO. One-Way ANOVA, $F(2/13)=4.769$, *$P=0.0284$ (Bonferroni multiple comparison test), values are means±s.e.m, n=5.

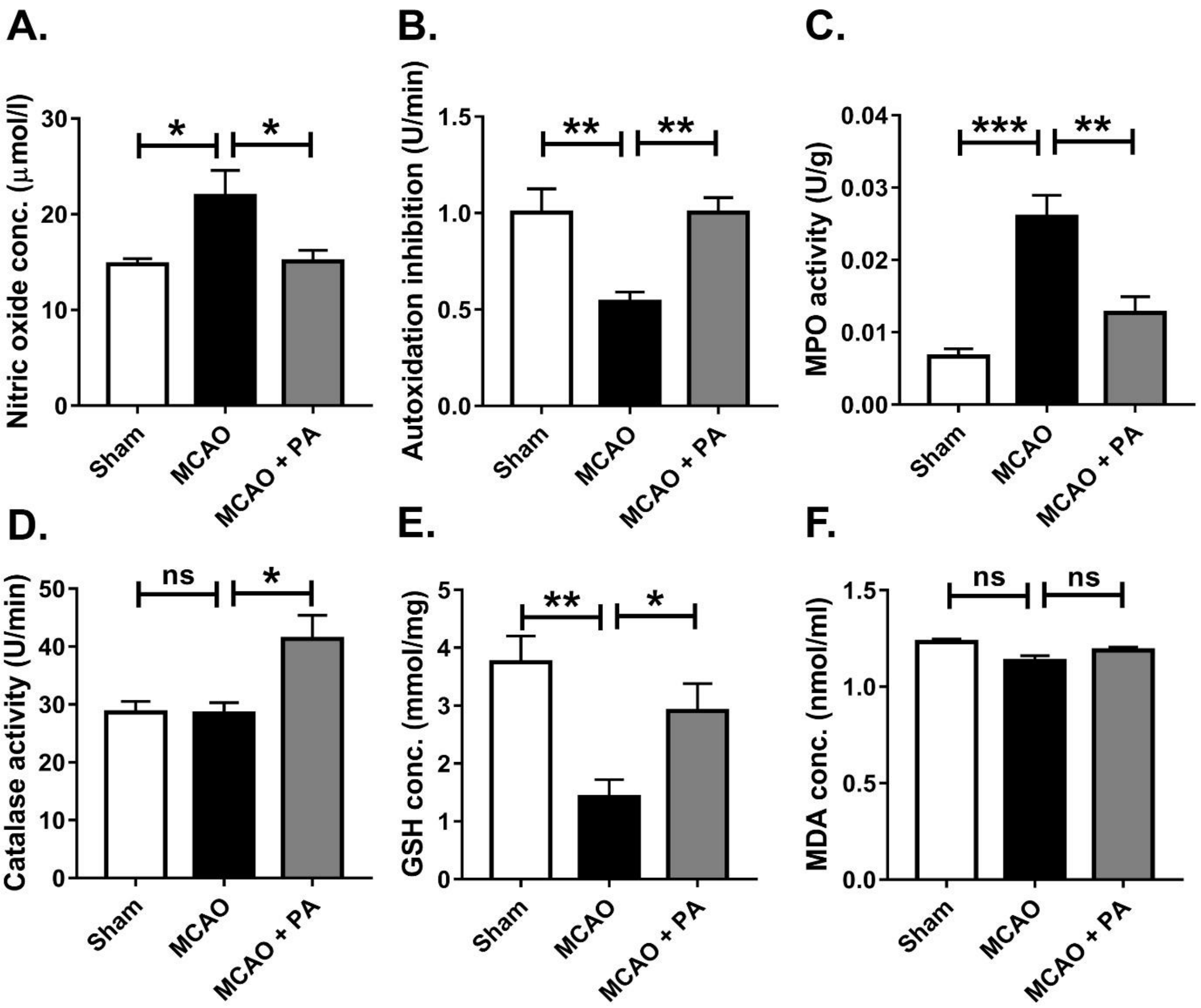

Figure 2: PA treatment reduces oxidative stress in the ischemic hemisphere 48 h after the MCAO.

A. PA treatment reduced the nitric oxide concentration in the ischemic hemisphere 48 h after the MCAO. The One-Way ANOVA, $F_{(2/12)}$=5.53, *P=0.0364 to 0.0464 (Bonferroni multiple comparison test), values are means±s.e.m, n=5. B. PA treatment increased autoxidation inhibition in the ischemic hemisphere 48 h after the MCAO. The One-Way ANOVA, $F_{(2/12)}$=10.56, **P=0.0040 to 0.0083 (Bonferroni multiple comparison tests), values are means±s.e.m, (n for Sham =4, n for MCAO= 5, n for MCAO+PA=6). C. PA treatment reduced

myeloperoxidase (MPO) activity in the ischemic hemisphere 48 h after the MCAO. The One-Way ANOVA, $F_{(2/11)}$=16.16, **P=0.0048, ***P=0.0005 (Bonferroni multiple comparison test), values are means±s.e.m,(n for Sham =4, n for MCAO= 4, n for MCAO+PA=6).

D. PA treatment increased the catalase activity in the ischemic hemisphere 48 h after the MCAO. The One-Way ANOVA, $F_{(2/12)}$=6.875, *P=0.0214, (Bonferroni multiple comparison test), values are means±s.e.m, n=5. E. PA treatment increased the GSH activity in the ischemic hemisphere 48 h after the MCAO. The One-Way ANOVA, $F_{(2/17)}$=9.232, *P=0.0484, **P=0.0019 (Bonferroni multiple comparison test), values are means±s.e.m, (n for Sham =6, n for MCAO= 8, n for MCAO+PA=6). F. PA treatment The effect of PA on malondialdehyde (MDA) concentration in the ischemic brain 48 h after the MCAO was insignificant. The One-Way ANOVA, $F_{(2/12)}$=3.981, P>0.05 (Bonferroni multiple comparison tests), values are means±s.e.m, (n for Sham =4, n for MCAO= 5, n for MCAO+PA=6).
